# Selenium Attenuates Chronic Heat Stress-Induced Apoptosis via the Inhibition of Endoplasmic Reticulum Stress in Mouse Granulosa Cells

**DOI:** 10.3390/molecules25030557

**Published:** 2020-01-28

**Authors:** Yongjie Xiong, Qirun Yin, Erhui Jin, Huatao Chen, Shaojun He

**Affiliations:** 1 College of Animal Science, Anhui Science and Technology University, Fengyang, Anhui 233100, China; xiongyj@ahstu.edu.cn (Y.X.);; 2 Key Laboratory of the Quality and Safety Control for Pork of the Ministry of Agriculture, Anhui Science and Technology University, Fengyang, Anhui 233100, China; 3 Department of Clinical Veterinary Medicine, College of Veterinary Medicine, Northwest A&F University, Yangling, Shaanxi 712100, China; 4 Key Laboratory of Animal Biotechnology of the Ministry of Agriculture, College of Veterinary Medicine, Northwest A&F University, Yangling, Shaanxi 712100, China

**Keywords:** selenium, heat stress, endoplasmic reticulum stress, apoptosis, granulosa cells

## Abstract

Heat stress induces apoptosis in various cells. Selenium, an essential micronutrient, has beneficial effects in maintaining the cellular physiological functions. However, its potential protective action against chronic heat stress (CHS)-induced apoptosis in granulosa cells and the related molecular mechanisms are not fully elucidated. In this study, we investigated the roles of selenium in CHS-induced apoptosis in mouse granulosa cells and explored its underlying mechanism. The heat treatment for 6–48 h induced apoptosis, potentiated caspase 3 activity, increased the expression levels of apoptosis-related gene BAX and ER stress markers, glucose-regulated protein 78 (GRP78), and CCAAT/enhancer binding protein homologous protein (CHOP) in mouse granulosa cells. The treatment with ER stress inhibitor 4-PBA significantly attenuated the adverse effects caused by CHS. Selenium treatment significantly attenuated the CHS- or thapsigargin (Tg, an ER stress activator)-induced apoptosis, potentiation of caspase 3 activity, and the increased protein expression levels of BAX, GRP78, and CHOP. Additionally, treatment of the cells with 5 ng/mL selenium significantly ameliorated the levels of estradiol, which were decreased in response to heat exposure. Consistently, administering selenium supplement alleviated the hyperthermia-caused reduction in the serum estradiol levels in vivo. Together, our findings indicate that selenium has protective effects on CHS-induced apoptosis via inhibition of the ER stress pathway. The current study provides new insights in understanding the role of selenium during the process of heat-induced cell apoptosis.

## 1. Introduction

Heat stress is a non-specific response to the thermal environment, which is known to be a major risk factor for diminishing reproductive efficiency and performance usually in female mammals during summer [1,2]. The existing evidence shows that heat stress causes abnormal follicular atresia, steroid hormone secretion disorder in the ovary, and even female infertility [3]. As compared to acute heat stress, the intensity of these effects is relatively weak in chronic heat stress. Nonetheless, the duration of chronic heat stress is relatively long, which results in severe economic loss in the livestock industry [4]. Over recent decades, although there has been an increasing number of investigations that had focused on heat stress-associated ovarian injury, its precise molecular mechanism remains poorly understood especially in relation to chronic heat stress. It is well known that granulosa cell apoptosis is an important marker and inducer of follicular atresia, and it plays critical roles in sustaining the normal physiological functions of the ovary, such as follicular growth and hormone synthesis [5]. Previous studies have shown that heat stress inhibits proliferation and induces apoptosis of ovarian granulosa cells, which were closely related to ovarian dysfunction induced by heat stress in various species [6,7,8]. This implies that maintaining the normal physiological functions of granulosa cells under heat stress may be of benefit to prevent or attenuate the ovarian injury caused by heat stress.

In eukaryotes, endoplasmic reticulum (ER) is an important organelle for the folding, modification, and maturation of newly synthesized proteins. A number of pathological factors can disturb the balance between the protein loading and the folding capacity of the ER, which results in triggering the ER stress [9,10,11]. If the homeostasis of the ER microenvironment is not restored, severe or persistent ER stress eventually induces apoptosis [12]. It was reported that ER stress-mediated granulosa cell apoptosis plays an important role in the progression of follicular atresia through GRP78 and CHOP activation in the goat ovary [13]. Furthermore, it was reported that ER stress signaling pathway was activated by heat stress in HeLa and HepG2 cells in vitro [14]. Moreover, the ER stress signaling induced by testicular hyperthermia is responsible for the apoptosis of spermatocytes in the mouse testis [15]. Notably, increasing evidence has strongly suggested that ER stress is involved in various pathologies of reproductive diseases and heat-induced cell death.

Selenium (Se) is an essential nutritional trace element for the humans and animals, and it exists in soil mainly as a mineral sodium selenite [16]. It has been reported that the preventive and therapeutic effect of sodium selenite on cancer. This metalloid is believed to be antioxidant due to its presence in the active centers of reactive oxygen species (ROS) decomposing peroxidases and superoxide dismutases [17]. Because of its well-known function as an antioxidant, Se has been widely used for regulating the metabolic disorders and reproductive physiological functions [18]. Se has been documented to effectively protect certain cells against the toxicant and ER stress induced apoptosis [19]. Nevertheless, it is still not fully understood whether Se has a protective effect through the inhibition of ER stress signaling against heat stress-induced apoptosis in mouse granulosa cells.

The aim of this study was to explore the effects of Se on chronic heat stress-induced damage in granulosa cells using an in vitro mouse granulosa cell model, and to evaluate the potential mechanism by which Se attenuates cell apoptosis. Moreover, we evaluated the role of Se in controlling the estradiol secretion levels in the heat-stressed mouse granulosa cells and mice exposed to chronic heat stress. The current study may help to clarify the molecular mechanism related to the protective effects of Se against heat stress induced apoptosis in the granulosa cells, providing a basis for identifying the novel therapeutic strategies for ovarian injury related to hyperthermia.

## 2. Results

### 2.1. Heat Stress Induces Cell Apoptosis via the Activation of ER Stress in Mouse Granulosa Cells

**Figure 1:**
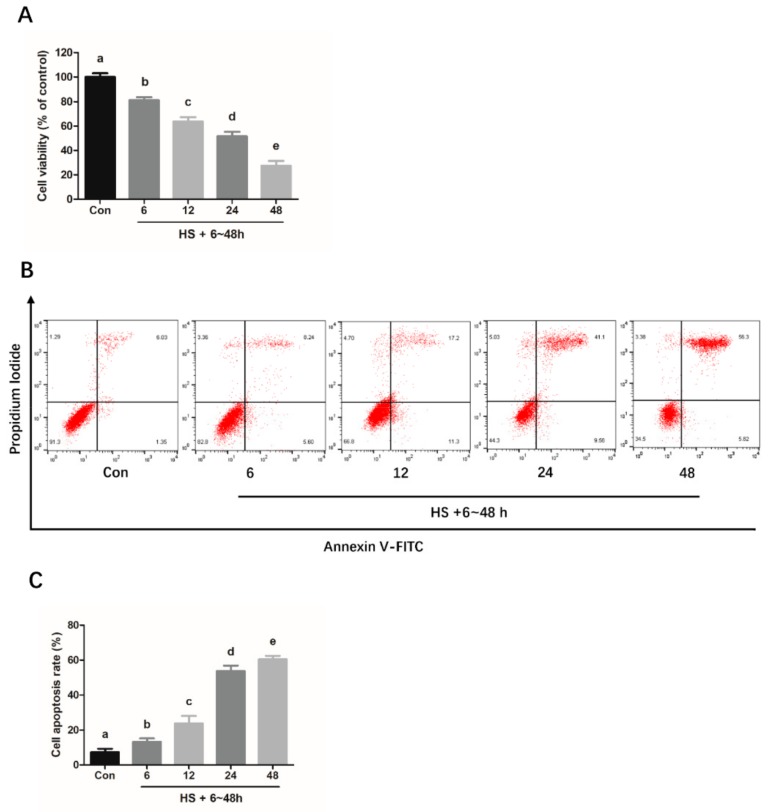

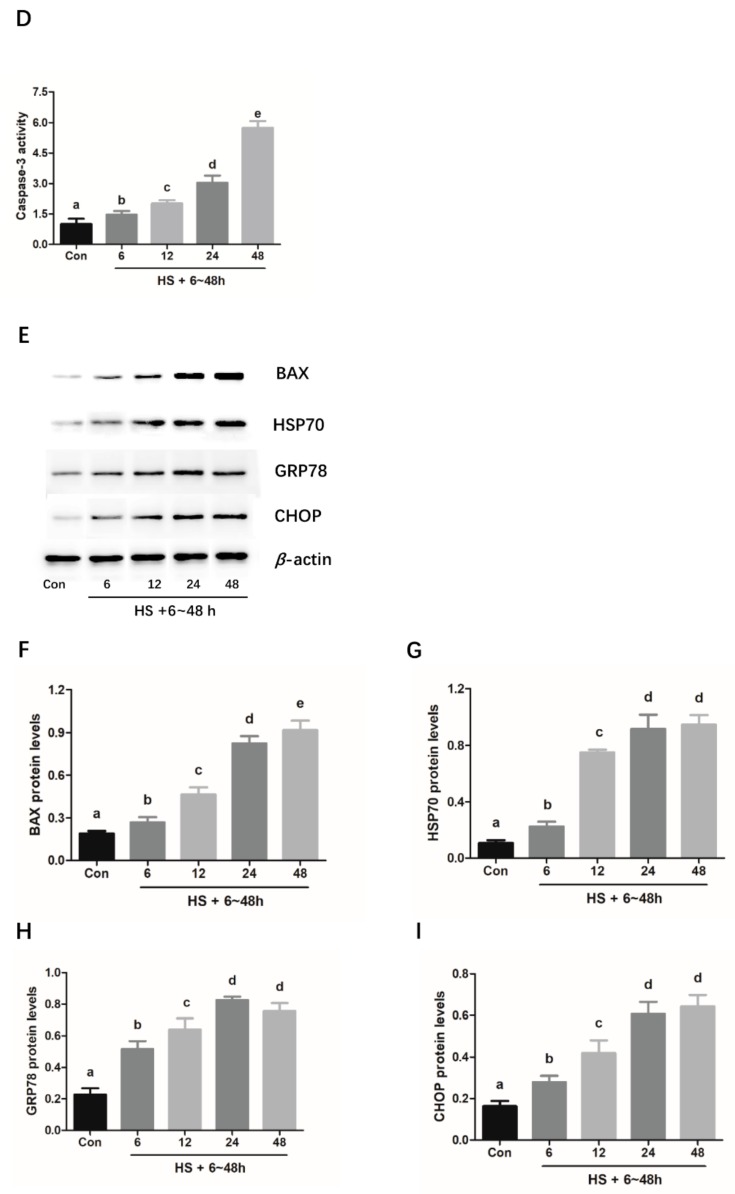
Chronic heat stress induces apoptosis and ER stress in mouse granulosa cells. Cells were cultured at 39 °C for heat stress for different periods of time (0, 6, 12, 24, and 48 h). Then, the treated cells were harvested for performing the CCK-8 assay (**A**) and flow cytometry-based analysis of apoptosis (**B** and **C**). Caspase-3 activity was measured by using a Caspase 3 Activity Assay Kit (**D**). Western blotting (**E**) was used to analyze the protein expression levels of cell apoptosis-related BAX (**F**), heat stress-related marker protein HSP70 (**G**) and ER stress activation markers, GRP78 (**H**) and CCAAT/enhancer binding protein homologous protein (CHOP) (**I**). β-actin was used for normalizing the level of protein expression. The results of data analysis are shown as the bar graphs. The data are presented as mean ± SEM of three independent experiments, and each independent experiment includes three technical replicates. Bars with different lowercase letters are significantly different (*p* < 0.05).

As shown in Figure 1A, the cell viability was significantly decreased following heat treatment for different time periods. To determine whether the reduced cell viability was due to the apoptosis induced by heat treatment, the heat-treated mouse granulosa cells were subjected to flow cytometry analysis, colorimetric assay, and western blotting. The results show that the cellular apoptotic rate and the expression levels of apoptosis-related genes (*Caspase 3* and *BAX*) increased in a time-dependent manner (Figure 1B–F). Similarly, the heat treatment of mouse granulosa cells for 6–24 h significantly increased the protein levels of HSP70, a key marker of heat stress, and ER stress-related proteins (GRP78 and CHOP) (Figure 1E,G–I). However, as compared to 24 h group, no significant differences were observed in the expression of HSP70, GRP78, and CHOP in 48 h group (Figure 1E,G–I).

### 2.2. Sodium Selenite Attenuates the Heat Stress-Induced Apoptosis and ER Stress in Mouse Granulosa Cells

**Figure 2:**
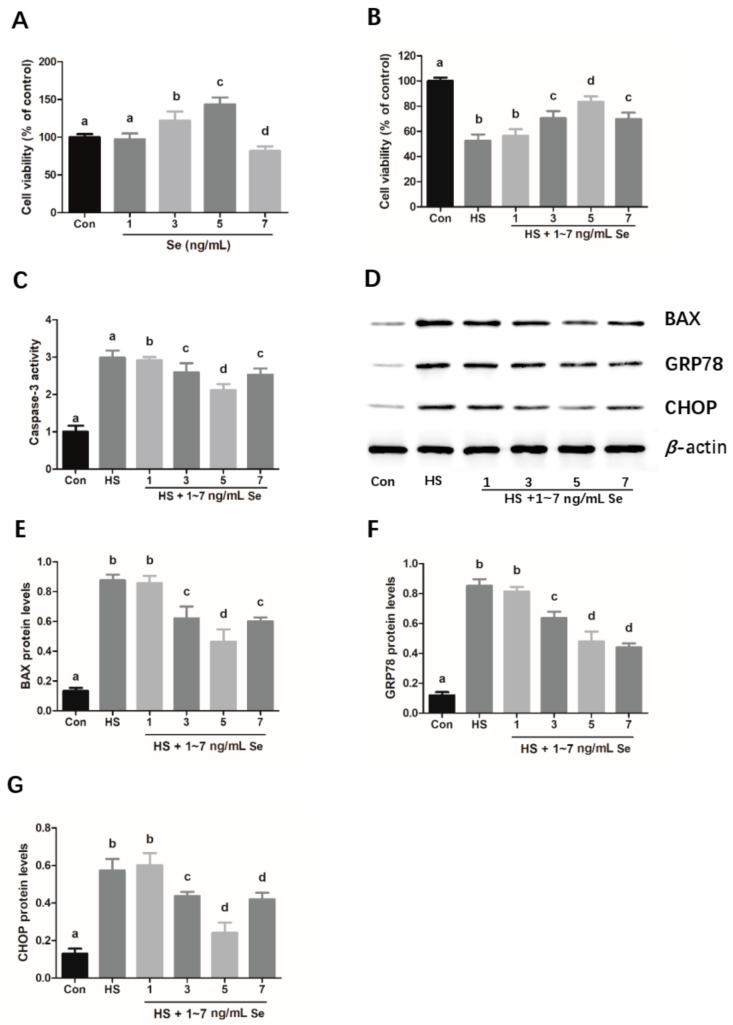
Sodium selenite attenuates the chronic heat stress-induced cell viability decreases and ER stress in mouse granulosa cells. Cells were treated with different concentrations of sodium selenite (1, 3, 5, and 7 ng/mL) at 37 °C (**A**) or at 39 °C (**B**) for 24 h, and then harvested for analyzing the cell viability by CCK-8 assay. Caspase-3 activity was analyzed using a Caspase 3 Activity Assay Kit (**C**). Western blot analysis of apoptosis-related protein BAX, ER stress activation marker GRP78 and CHOP are shown (**D**). The relative protein expression of BAX (**E**), GRP78 (**F**) and CHOP (**G**) were normalized to β-actin. The results of data analysis are shown as the bar graph. The data are presented as mean ± SEM of three independent experiments, and each independent experiment includes three technical replicates. Bars with different lowercase letters are significantly different (*p* < 0.05).

To investigate the effect of Se on mouse granulosa cell viability, mouse granulosa cells were treated with different concentrations of sodium selenite (1, 3, 5, and 7 ng/mL) for 24 h. As shown in Figure 2A, 1 ng/mL sodium selenite had no effect on the viability of mouse granulosa cells, whereas sodium selenite significantly increased the cell viability in the 3 ng/mL and 5 ng/mL group, as compared to the control cell group. Simultaneously, the cells treated with 7 ng/mL sodium selenite showed significantly decreased cell viability (Figure 2A). Furthermore, the decreased cell viability due to heat treatment was effectively restored in response to 5 ng/mL sodium selenite (Figure 2B). At the same time, 5 ng/mL sodium selenite was revealed to obviously inhibit caspase 3 activity and the protein expression levels of BAX protein (Figure 2C–E). Additionally, the heat stress induced upregulation of the expression levels of GRP78 and CHOP was significantly suppressed by treatment with 5 ng/mL sodium selenite (Figure 2D,F–G). Interestingly, the cell viability of 7 ng/mL sodium selenite treated group was lower than the 5 ng/mL sodium selenite treated group but higher than the heat stress-treated group (Figure 2B). Consistently, the caspase 3 activity and protein expression levels of BAX and CHOP in the 7 ng/mL sodium selenite treated group were higher than the 5 ng/mL sodium selenite treated group (Figure 2C–E,G). However, there was no significant difference in the GRP78 expression levels between the 5 ng/mL and 7 ng/mL sodium selenite treated groups (Figure 2D,F).

### 2.3. 4-Phenylbutyrate (4-PBA) Attenuates the Heat Stress-Induced Apoptosis and ER Stress in Mouse Granulosa Cells

**Figure 3:**
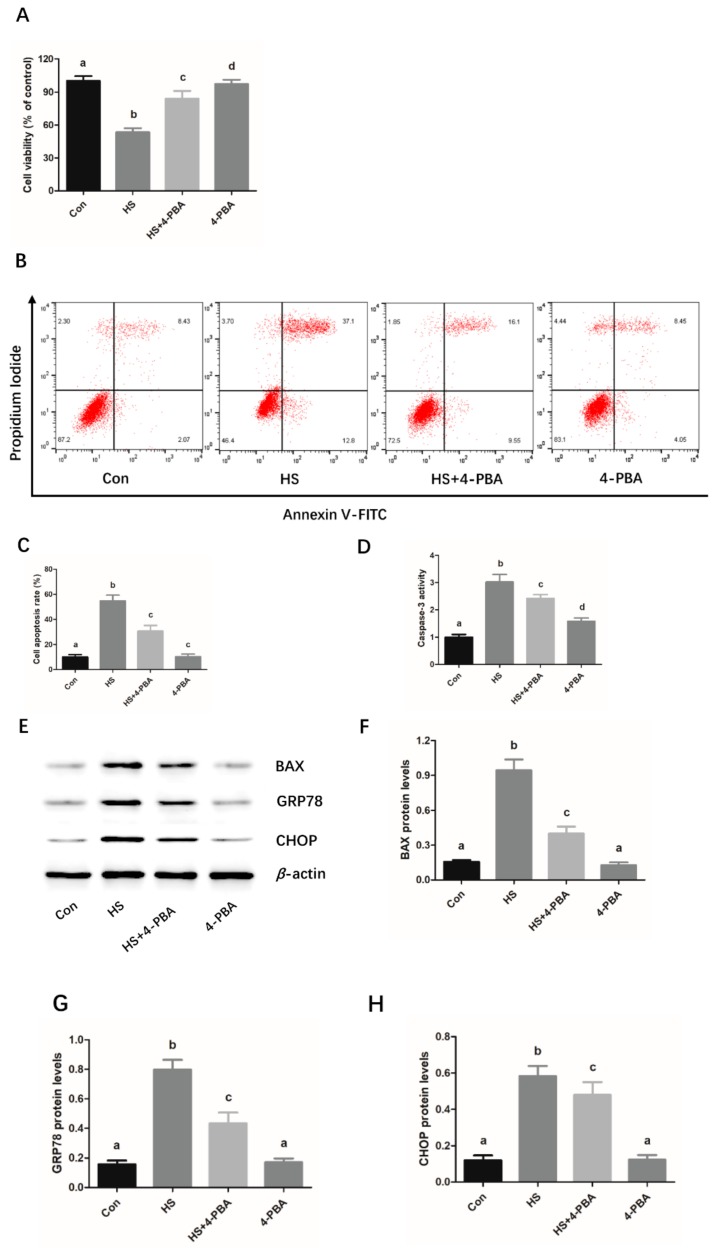
4-PBA attenuates the heat stress-induced apoptosis and ER stress in mouse granulosa cells. Cells were treated with or without 4-PBA (500 nM) at 39 °C for 24 h, and then harvested for analyzing the cell viability and apoptotic rate by CCK-8 assay (**A**) and flow cytometry (**B**, **C**), respectively. Caspase 3 Activity of the mouse granulosa cells was analyzed by a colorimetric assay kit (**D**). Western blot analysis of the expression of BAX, GRP78, and CHOP are shown (**E**). The relative protein expression levels of BAX (**F**), GRP78 (**G**) and CHOP (**H**) were normalized to β-actin. The statistical analysis results are shown as bar graphs. The data are represented as the mean ± SEM of three independent experiments, and each independent experiment includes three technical replicates. Bars with different lowercase letters are significantly different (*p* < 0.05).

The data from the CCK-8 assay and flow cytometry indicated that heat stress treatment significantly decreased the cell viability and induced cell apoptosis, whereas treatment with 4-PBA, an ER stress inhibitor, markedly restored the cell viability and reduced apoptosis (Figure 3A–C). Moreover, it was observed that 4-PBA treatment not only significantly inhibited the caspase 3 activity, but also reduced the expression levels of BAX, GRP78, and CHOP in the heat stress-treated mouse granulosa cells (Figure 3D–H).

### 2.4. Sodium Selenite Protects the Cells Against Thapsigargin (Tg)-Induced Cytotoxicity, Apoptosis, and ER Stress in Mouse Granulosa Cells

**Figure 4:**
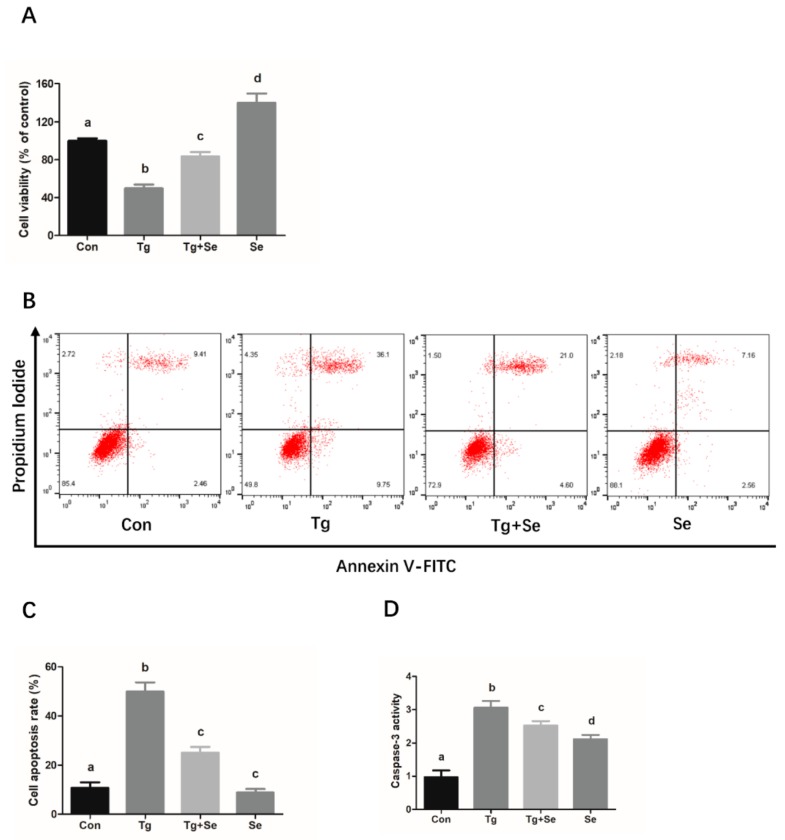

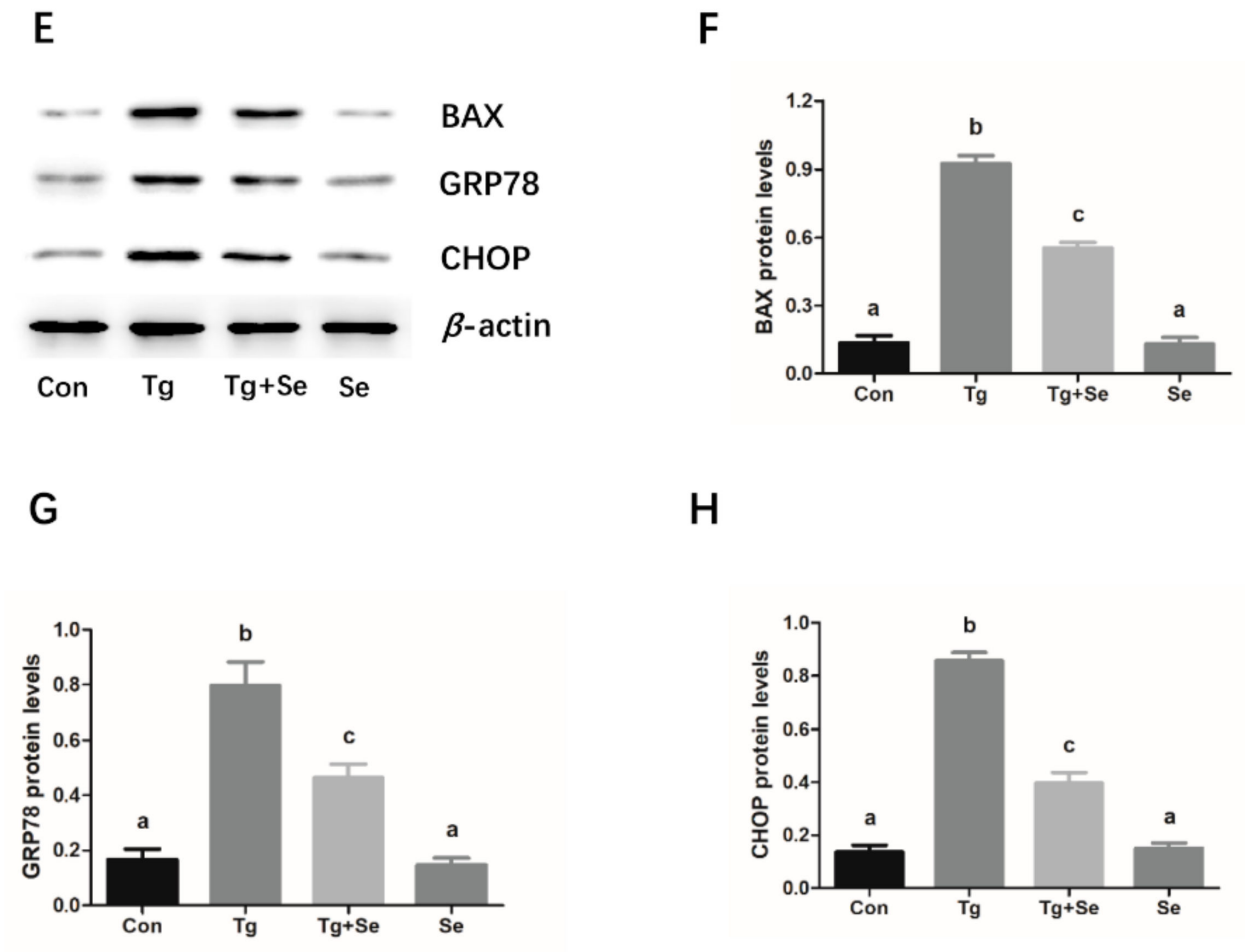
Sodium selenite attenuates Tg-induced apoptosis and ER stress in mouse granulosa cells. Cells were treated with Tg (500 nM) in the absence or presence of sodium selenite (5 ng/mL) at 37 °C for 24 h, and then processed for cell viability and apoptosis analysis by CCK-8 kit (**A**) and flow cytometry (**B**, **C**), respectively. Caspase 3 Activity of the MGCs is measured by a colorimetric assay kit (**D**). Western blotting (**E**) was used to analyze the protein expression of BAX (**F**), GRP78 (**G**), and CHOP (**H**). β-actin was used to normalize the protein expression levels. The statistical analysis results are shown as bar graphs. The data are represented as the mean ± SEM of three independent experiments, and each independent experiment includes three technical replicates. Bars with different lowercase letters are significantly different (*p* < 0.05).

It was observed that Tg, an ER stress agonist, induced a decrease in the mouse granulosa cell activity and increased the expression levels of ER stress markers, GRP78 and CHOP, while enhancing the apoptosis-related caspase 3 activity and BAX expression. Meanwhile, the cells treated with 5 ng/mL sodium selenite significantly inhibited Tg-caused activity decreases and attenuated Tg-induced ER stress and apoptosis (Figure 4).

### 2.5. Sodium Selenite Ameliorates Heat Stress-Induced Reduction of Estradiol Production in Mouse Granulosa Cells

**Figure 5:**
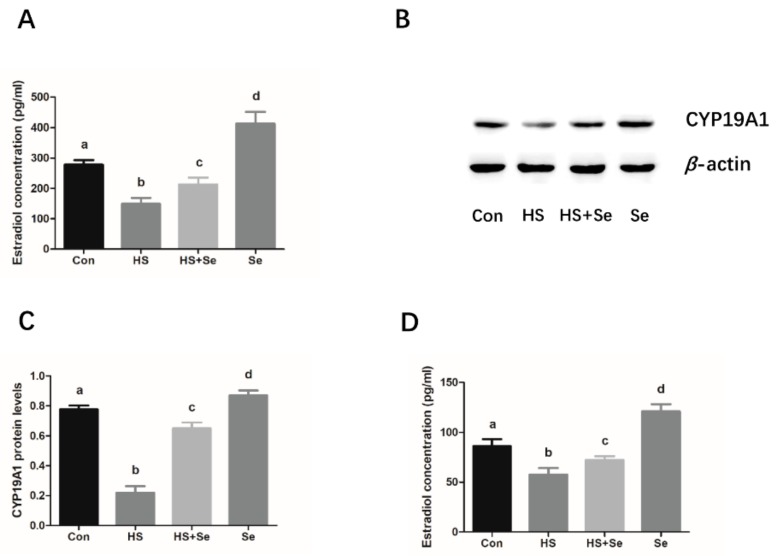
Effects of sodium selenite on estradiol levels in heat stress-treated granulosa cells and serum in mouse. Cells were treated with or without sodium selenite at 39 °C for 24 h and then the concentration of estradiol in the culture supernatants was detected by an ELISA kit (**A**). Western blotting was used to analyze the protein expression of CYP19A1 (**B** and **C**). β-actin was used to normalize the protein expression levels. The mouse was orally administered sodium selenite for 4 weeks, and the estradiol level in the serum was detected by an ELISA kit (**D**). The statistical analysis results are shown as bar graphs. The data are represented as the mean ± SEM of three independent experiments, and each independent experiment includes three technical replicates. Bars with different lowercase letters are significantly different (*p* < 0.05).

As shown in Figure 5A, as compared to the control cell group, the concentration of estradiol was significantly decreased in the heat stress treated cells, whereas sodium selenite significantly inhibited the reduction of estradiol production in the mouse granulosa cells treated with heat stress. Moreover, sodium selenite was revealed to significantly increase the protein expression levels of CYP19A1, which is a rate-limiting enzyme for the estradiol synthesis (Figure 5B,C). Subsequently, we investigated the effects of sodium selenite on serum estradiol levels in the mice exposed to heat stress. The data shows that serum hormone levels were abnormal in mice that suffered from heat stress, which was exhibited as a decrease in the serum levels of estradiol. However, the reduced estradiol concentration in the serum was significantly ameliorated in mice supplied with sodium selenium (Figure 5D).

## 3. Discussion

Se is an essential trace mineral that has a variety of beneficial effects on animal and human health, such as antioxidative effects, antitumor effects, and reproductive function regulation [20,21]. Therefore, several studies were focused on the effects of Se in different physiological and pathological processes [22,23]. In the present study, we have shown that chronic heat stress had adverse effects on the mouse granulosa cells including the reduction in cell viability, induction of apoptosis, potentiation of caspase 3 activity, and upregulation of the expression of apoptosis-related crucial protein BAX and ER stress activation markers, GRP78 and CHOP. Sodium selenite significantly inhibited the chronic heat stress-induced reduction in cell viability and increased the protein expression levels of apoptosis-related genes and ER stress activation markers. Furthermore, sodium selenite inhibited the chronic heat stress-induced reduction in estradiol production in the granulosa cells in vitro and in vivo.

A prior study has reported that the expression levels of caspase-3 are upregulated, and that the mitochondrial apoptotic pathway is involved in the acute heat stress-induced apoptosis in the mouse granulosa cells that suffer the high temperature treatment [24]. However, the molecular mechanism underlying the chronic heat stress-induced apoptosis in mouse granulosa cells is still not well understood. Moreover, it has been documented that the caspase-3 activity, as an executioner in caspase-mediated cell death, is positively in line with the cell apoptosis rate in the human granulosa cells [25], and BAX not only has a pro-apoptotic effect, but also plays an important role in apoptosis in rat granulosa cells [26]. Therefore, to explore the underlying mechanism of chronic heat stress-induced apoptosis in granulosa cells, we first determined the cell viability and apoptotic rate of cells that suffered chronic heat stress for different periods of time. In this study, chronic heat stress was found to not only reduce the cell activity and induce apoptosis, but also enhanced the caspase 3 activity and BAX protein expression levels in a time-dependent manner in the granulosa cells, which is in agreement with the prior report [24]. Therefore, we speculated that the potentiation of caspase-3 activity and increased BAX protein expression levels may be responsible for the chronic heat stress-induced apoptosis in mouse granulosa cells. Moreover, previous studies have indicated that the ER stress-mediated apoptotic pathway participated in heat stress-induced apoptosis in various cells, including human osteosarcoma cells and bovine granulosa cells [27,28]. To examine whether ER stress is implicated in the chronic heat stress-induced mouse granulosa cell apoptosis, we analyzed the protein levels of ER stress markers, GRP78 and CHOP. In this study, a significant time-dependent increase in the protein expression levels of GRP78 and CHOP in the granulosa cells after heat treatment for 6–24 h was observed, suggesting that ER stress was activated and was closely related to the process of apoptosis. Based on these findings, we speculated that the suppression of ER stress in the granulosa cells may beneficially attenuate the adverse effects of chronic heat stress. Simultaneously, to determine the degree of heat stress, we examined the HSP70 protein expression levels at different timepoints after heat treatment. Western blotting demonstrated that the protein expression levels of HSP70 gradually increased after 6–24 h of treatment at 39 °C, suggesting that 39 °C heat treatment can successfully induce heat stress in mouse granulosa cells. Interestingly, HSP70 expression levels showed no significant difference between the 24 h group and 48 h group. It is well established that HSP70, the most heat stress inducible protein, not only is a standard index for heat stress activation, but also plays a key role in the process of heat tolerance by maintaining cellular homeostasis [29,30]. HSP70 has a protective effect on the cells against heat stress-induced apoptosis-initiated caspase 3 activation [31]. In other words, an increase in HSP70 expression often indicates an increase in the intensity of heat stress. Furthermore, the flow cytometry data showed that the cell apoptotic rate was over 60%, implying that the intensity of heat treatment (48 h) may have been too long to go beyond the tolerance of heat in the mouse granulosa cells, and the normal physiological function of most granulosa cells may have been severely damaged after 48 h of heat treatment due to prolonged exposure to heat stress.

Se has been shown to play a significant protective role in the inhibition of caspase-3 and BAX-mediated apoptosis caused by various pathological factors [32,33]. In the present study, mouse granulosa cells were used as the model system to examine the protective effects of sodium selenite on chronic heat stress-induced granulosa cell apoptosis and its underlying mechanism. Firstly, the effects of sodium selenite on the cell viability and apoptosis of mouse granulosa cells were investigated. The results show that relatively low dosages of sodium selenite (3 ng/mL and 5 ng/mL) could alleviate the heat stress-induced reduction of cell viability and increased the protein expression levels of apoptosis-related key modulators, including caspase 3 and BAX. Based on these findings, it could be inferred that sodium selenite inhibited the apoptosis of mouse granulosa cells caused by chronic heat stress, with 5 ng/mL sodium selenite providing greater protection. However, it was observed that the cell viability was significantly decreased in the group treated with 7 ng/mL sodium selenite, implying that sodium selenite at certain high concentrations may have cytotoxic effects on the granulosa cells. It is not surprising to observe cytotoxicity in response to high levels of Se as high concentration of Se can significantly inhibit cell growth in some cell types, such as glioblastoma and mouse brain metastatic cells [34,35]. Simultaneously, it has been demonstrated that the form of Se supplemented as organo-Se compounds or in the form of inorganic Se has an important bearing on its potential beneficial and/or untoward toxic effects on the health and growth of organism [36]. Although the cytotoxicity of Se is observed in the group treated with 7 ng/mL Se (sodium selenite), whether the same dose of organic Se, such as selenomethionine or selenised yeast, has a cytotoxic effect on mouse granulosa cells remains unclear and needs further investigation. Furthermore, Se also has been documented to decrease the apoptosis of immune cells by suppressing ER stress in chickens [19]. The CHOP/ caspase-3 apoptotic signaling pathway has been implicated in the inhibitive effects of Se on lead-induced apoptosis via ER stress pathway in chicken testes [37], indicating that ER stress-mediated apoptosis pathway plays an important role in the protective effects of selenium against apoptosis induced by different pathological factors. In the present study, our data indicated that sodium selenite suppressed the chronic heat stress-induced increase in the protein expression levels of GRP78 and CHOP, suggesting that sodium selenite might attenuate heat stress-induced cell impairment and ER stress in mouse granulosa cells. In addition, to determine the role of ER stress in heat stress-induced apoptosis, an ER stress inhibitor 4-PBA was utilized. We found that 4-PBA treatment significantly prevented cell apoptosis caused by heat stress. Simultaneously, we found that sodium selenite could protect the granulosa cells against the decreased cell viability, cell death, and ER stress caused by the ER stress activator Tg. These findings further verified that ER stress is involved in the process of chronic heat stress-induced mouse granulosa cell apoptosis, and sodium selenite is beneficial for protecting the mouse granulosa cells against Tg or chronic heat stress-induced cell injury via the suppression of ER stress response.

Estradiol is as an important steroid hormone that is mainly produced by the ovary and plays an essential role in maintaining the reproductive function including ovarian folliculogenesis, maturation, and ovulation in the ovary [38]. It can also stimulate granulosa cell proliferation and regulate the expression of gonadotrophin receptors in the granulosa cells [39]. Previous studies have suggested that heat stress significantly reduced the estradiol levels in the ovary in goats, cows, and mice [24,40,41]. The diminished estradiol production caused by heat stress might result in enhanced susceptibility to apoptosis in rat granulosa cells [42]. Furthermore, it is worth noting that that Se exerts a stimulatory effect on the estradiol production in bovine granulosa cells and goat luteinized granulosa cells [43,44]. To further confirm the protective role of Se in inhibiting cell injury caused by chronic heat stress in granulosa cells, we investigated the effects of sodium selenite on estradiol levels in mouse granulosa cells after exposing the cells to high temperature. As expected, the reduced estradiol production was observed in the mouse granulosa cells that were exposed to chronic heat stress, while sodium selenite effectively increased the estradiol levels. The protein expression levels of CYP19A1, a rate-limiting enzyme for estradiol synthesis from the aromatization of androgen [45], were upregulated in mouse granulosa cells after sodium selenite treatment. Thus, we infer that sodium selenite diminishes the reduction of estradiol production in heat stress-treated mouse granulosa cells, which may be closely associated with the increased CYP19A1 expression levels. Subsequently, we further explored the protective effects of sodium selenite on estradiol production in hyperthermia-induced heat stress in female mice. Similar to the prior reports, we found that the estradiol levels were significantly downregulated in the mice exposed to cyclic hyperthermia environment. However, it is noteworthy that sodium selenite supplements could effectively restore the reduced serum levels of estradiol in mice suffering from hyperthermia treatment, thus, implying that sodium selenite had a protective effect against the reduction of estradiol level in mice that suffered hyperthermia.

Additionally, a major factor that can affect serum estradiol levels and/or ovary is the number of granulosa cells. In the normal physiological process in the ovary, a certain degree of cell death can be observed in the granulosa cells, which plays an important role in accurately modulating the discarded decrepit cells, thereby maintaining the self-renewal of the granulosa cell population and normal estradiol levels [46,47]. However, excessive or severe granulosa cell death induced by damage to ovary usually results in reduced estradiol levels, ultimately leading to female reproductive dysfunction [48]. In a prior study, Wang et al. found that suppressing the extracellular signal-regulated kinase 1/2 (ERK1/2) signaling pathway was shown to effectively inhibit the decreased apoptotic rate, which restored the decreased estradiol levels of bovine granulosa cells under high temperature [49]. Similar to this study, the heat treatment was displayed to activate the ER stress to reduce the number of normal mouse granulosa cells and affect the normal estradiol secretion levels of these cells, and sodium selenite had an inhibitory effect on the enhanced ER stress caused by high temperature to decrease the granulosa cell apoptotic rate. Taken together, these data indicate that sodium selenite attenuates heat stress-caused decreases of estradiol levels, which may be closely related to the reduced ER stress-mediated apoptosis through the suppression of ER stress and increase in the CYP19A1 levels in mouse granulosa cells.

In the previous decade, there has been a growing number of investigations focusing on the mechanism of the protective effect of selenium against various stress in different cells. In diseased macrophages by accumulation of esterified cholesterol, CHOP associated with stress in the ER activates the 3-phosphatidyl inositol receptor that opens the calcium channel in ER, causing calcium to be released into the cytoplasm. Increased calcium ions activate calcium and calmodulin dependent proteinase II (CaMKII), affecting the induction of the mitochondrial apoptosis pathway [50,51]. Moreover, it is well established that the Se inhibits apoptosis via a mitochondrial-mediated pathway [52]. Furthermore, it is worth noting that most stress conditions, such as deficiency of growth factors, heat shock, and ER stress, also leads to disruption of the activity of the eIF2 initiating factor by phosphorylation of its α subunit. Phosphorylation eIF2α results in the formation of a stable eIF2-GDP complex with eIF2B, which strongly reduces the ability to exchange nucleotides guanine by eIF2B [53,54]. And it has been demonstrated that down-regulation of PERK-eIF2α-ATF4-CHOP pathway inhibits ER stress-mediated apoptosis [55]. In this study, although Se alleviates chronic heat stress-induced apoptosis via the inhibition of ER stress pathway in mouse granulosa cells, whether the PERK-eIF2α-ATF4-CHOP pathway is involved in this process remains unclear and needs further investigation.

In conclusion, our results reveal that chronic heat stress induces apoptosis in the granulosa cells through the ER stress signaling pathway. Moreover, sodium selenite could rescue these cells from chronic heat stress-caused apoptosis and ameliorate their estradiol production. These findings suggest that selenium may be used as a potential therapeutic agent for restoring heat stress associated reproductive dysfunction in females.

## 4. Materials and Methods

### 4.1. Animal Care and Treatment

The Kunming White outbred strain female mice were purchased from Jinan Pengyue Laboratory Animal Breeding Co. Ltd., Anhui branch (Hefei, China), and were housed in an environmentally controlled artificial room at 24–26 °C, where 12:12-h dark:light cycles were maintained with free access to feed and water. Then, the 21-day-old mice were sacrificed for the collection and culture of granulosa cells in vitro, and the 8-week-old mice were used for the experiment in vivo. All the experimental protocols were approved by the Committee for the Ethics on Animal Care and Experiments of Anhui Science and Technology University.

### 4.2. Collection, Culture, and Heat Treatment of Mouse Granulosa Cells

The granulosa cells were collected from the mouse ovary and cultured according to the procedure described in our prior report [56]. Briefly, the 21-day-old female mice were administered 6 IU pregnant mare serum gonadotropin (PMSG) by intraperitoneal injection before collecting the ovary. After 46 h of injection, the 26-gauge needles were used to puncture the follicles to release the granulosa cells under germ-free conditions. The granulosa cells were filtered and pelleted by centrifugation. Following the analysis of cell viability, the granulosa cells were seeded into a 35-mm cell culture dish (1 × 10^5^/dish). Then, these cells were cultured in a mixed medium including 1% penicillin (HyClone, GE Healthcare Bio-Sciences Corp., Piscataway, NJ, USA), 1% streptomycin (HyClone, GE Healthcare Bio-Sciences Corp., Piscataway, NJ, USA), 10% FBS (Gibco, Life Technologies, Grand Island, NY, USA), and 88% DMEM/F12 medium (HyClone, GE Healthcare Bio-Sciences Corp., Piscataway, NJ, USA) at 37 °C under 5% CO_2_. When the confluence of cells was approximately 80%, all cells were switched to a 39 °C cell incubator in 5% CO_2_ for chronic heat stress experiments as described in a prior study, with minor modification [57]. In brief, after pre-culturing for nearly 48 h, the cells were cultured in fresh medium at 39 °C for 0, 6, 12, 24, and 48 h to induce chronic heat stress. Then, the cells were harvested for CCK-8 assay, flow cytometry, caspase 3 activity analysis, and western blotting.

To examine the effect of sodium selenite on cell viability, sodium selenite (Sigma-Aldrich, St. Louis, MO, USA) at different concentrations (1, 3, 5, and 7 ng/mL) was added into the medium at 37 °C or 39 °C for 24 h, and the cells without sodium selenite in the medium served as controls. For 4-Phenylbutyrate (4-PBA, an ER stress antagonist, Sigma-Aldrich, St. Louis, MO, USA) treatment experiments, the cells were treated with or without 4-PBA (500 nM) at 39 °C for 24 h. In the thapsigargin (Tg, an ER stress activator, Sigma-Aldrich, St. Louis, MO, USA) treatment assay, the cells were treated with Tg (500 nM) in the absence or presence of sodium selenite (5 ng/mL) at 37 °C for 24 h. At last, all the cells were collected for performing the CCK-8 assay, flow cytometry, caspase 3 activity analysis, and western blotting.

### 4.3. Analysis of Cell Viability

To determinate the adverse effects of chronic heat stress on mouse granulosa cells, the cells cultured at 39 °C for 0, 6, 12, 24, and 48 h were collected, and the cell viability was detected using a commercial CCK-8 assay kit (Solarbio, Beijing Solarbio Science & Technology, Beijing, China) according to the procedure described in a prior report [58]. Briefly, mouse granulosa cells were plated into the 96-well plates (3 × 10^4^ cells per well). After cultivation at 39 °C for different time periods, 0, 6, 12, 24, and 48 h, 10 μL CCK-8 solution was added to each well, and then, the 96-well plates were again incubated at 37 °C for 2 h. Finally, the absorbance was detected using a 96-well plate reader (ThermoFisher Scientific, Vantaa, Finland) at 450 nm.

### 4.4. Analysis of Cell Apoptotic Rate

The cell apoptotic rate was analyzed using an Annexin V-FITC/PI apoptosis detection kit (KeyGen, Nanjing KeyGen Biotech, Nanjing, China) after collection of the cells according to the procedure described in a prior report [59]. The cells were washed with PBS, centrifuged, and were suspended in 500 μL binding buffer in a microtube; then, 5 μL each of the Annexin V- FITC and PI staining solutions were added and mixed with the cells. Finally, the mixture was incubated away from light at room temperature for 15 min and was analyzed using BD FACSCaliburTM Flow Cytometer (BD Biosciences, San Jose, CA, USA) within 1 h. The total of Annexin V-FITC^+^/PI^-^ cells and Annexin V-FITC^+^/PI^+^ cells was considered as apoptotic cells.

### 4.5. Analysis of Caspase 3 Activity

The caspase 3 activity analysis was performed using a commercial caspase 3 activity assay kit (Beyotime, Beyotime Biotechnology, Shanghai, China) according to the procedure described in a prior study [60]. The harvested cells were centrifuged, washed with PBS buffer, and were mixed with cold lysis buffer for 15 min. After the cell mixture was centrifuged at 16,000× *g* for 10 min at 4 °C, the liquid supernatant was collected and 10 μL caspase 3 substrate (acetyl-Asp-Glu-Val-Asp p-nitroanilide) was added to it followed by incubation in a 96-wells microplate at 37 °C for 90 min. Finally, the absorbance was measured at 405 nm by a spectrophotometer (ThermoFisher Scientific, Vantaa, Finland).

### 4.6. Western Blotting

The cell proteins were extracted using the whole cell lysis assay kit (KeyGen, Nanjing KeyGen Biotech, Nanjing, China), and the protein concentration was measured using BCA protein quantitation assay kit (Nanjing KeyGen Biotech, Nanjing, China). A total of 35 μg protein of each cell sample was separated on a 12% SDS-PAGE gel and then transferred onto the polyvinylidene difluoride membranes. The membranes were fully soaked in western blocking buffer (Beyotime, Beyotime Biotechnology, Shanghai, China) for 60 min and then incubated with BAX (1:2000; Proteintech, Wuhan, China), HSP70 (1:1000; Proteintech, Wuhan, China), GRP78 (1:1000; Proteintech, Wuhan, China), CHOP (1:1000; Proteintech, Wuhan, China), and CYP19A1 (1:1000; Beyotime, Beyotime Biotechnology, Shanghai, China) primary antibody overnight at 4 °C. On the second day, these treated blot membranes were incubated with HRP-conjugated secondary antibody (1:5,000; Beyotime, Beyotime Biotechnology, China) for 60 min at room temperature, respectively. At last, the immunoreactive bands were visualized using a Clarity™ Western ECL Substrate Kit (Bio-Rad Laboratories, Hercules, CA, USA) and were determined by FlourChem HD2 gel imaging and analysis system (ProteinSimple, Santa Clara, CA, USA), and the densitometry of immunoreactive bands was analyzed using Quantity One software 4.6.2 (Bio-Rad Laboratories, Hercules, CA, USA).

### 4.7. Analysis of Estradiol Levels

To examine the effect of Se on estradiol levels on the chronic heat stress-treated mouse granulosa cells, the mouse granulosa cells were incubated at 39 °C for 24 h with or without sodium selenite (5 ng/mL). The cell culture supernatants of each treatment were collected, and the estradiol concentration was measured using an Estradiol Enzyme-Linked Immunosorbent Assay Kit (Beyotime, Beyotime Biotechnology, Shanghai, China) following the manufacturer’s instructions. To examine the effect of Se on the serum estradiol levels in vivo experiment, two hundred mice (8-week-old) were randomly assigned into four groups (*n* = 50 in each group), including a control group (Con), chronic heat stress group (HS), chronic heat stress + sodium selenite group (HS + Se) and a sodium selenite group (Se). Sodium selenite was dissolved in ultra-pure water (Resistivity:18 MΩ*cm) sourced from an ultra-pure water purification system (Milli-Q Integral, Merck Millipore, France) and was administered orally by drinking (1.6 ppm Se (20 μM Na_2_SeO_3_) in purified water) and maintained for 4 weeks. During the whole in vivo treatment, the HS group and HS + Se group mice were exposed to a constant temperature of 39 °C for 8 h per day, while the Con group and Se group were housed at a room temperature of 24–26 °C. Following the treatment, the blood samples of all mice were collected for analyzing the estradiol levels by using an estradiol ELISA assay kit (Beyotime, Beyotime Biotechnology, Shanghai, China) according to the kit operating manual.

### 4.8. Statistical Analysis

The SPSS 17.0 (IBM SPSS Software, Armonk, NY, USA) was used to perform analysis of ANOVA, Fishers least significant differences, and independent sample *t*-tests. The data are shown as the mean ± SEM, and *p* < 0.05 represents statistically significant difference.

## Abbreviations

HS	Heat stress
ER	Endoplasmic reticulum
GRP78	Glucose-regulated protein 78
CHOP	CCAAT/enhancer-binding protein (C/EBP) homologous protein
CYP19A1	Cytochrome P450 family 19 subfamily A member 1
Se	Selenium
HSP70	Heat Shock Protein 70
PMSG	Pregnant Mare Serum Gonadotropin
4-PBA	4-Phenylbutyrate
Tg	Thapsigargin
